# 

**Published:** 1888-06-02

**Authors:** 


[THIRTY-SIX PAGES, INCLUSIVE OF EIGHT EXTRA.]

PRICE ONE PENNY.

No. 88, Vol. IV.—June 2nd, 1888.

[Registered at the General Post Office as a Newspaper.]

Per Annum, 6s. 6d., post free.

Monthly Parts, 6d.; post free, 7½d.

Ditto per Ann., 7s. 6d. post free.

### THE HOSPITAL

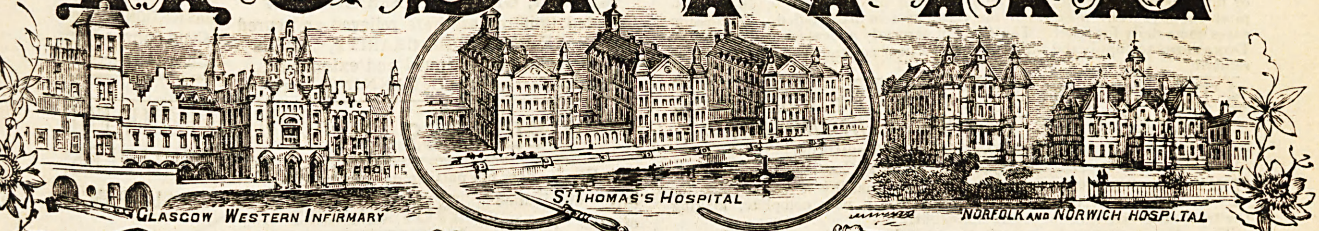

A WEEKLY INSTITUTIONAL JOURNAL OF

### Science, Medicine, Nursing, and Philanthropy.

### THE HOSPITAL SUNDAY FUND SPECIAL NUMBER.

